# Extraintestinal pathogenic *Escherichia coli* utilizes the surface-expressed elongation factor Tu to bind and acquire iron from holo-transferrin

**DOI:** 10.1080/21505594.2022.2066274

**Published:** 2022-04-20

**Authors:** Yu Sun, Xuhang Wang, Jin Li, Feng Xue, Fang Tang, Jianjun Dai

**Affiliations:** a MOE Joint International Research Laboratory of Animal Health and Food Safety, Key Lab of Animal Bacteriology, Ministry of Agriculture, College of Veterinary Medicine, Nanjing Agricultural University, Nanjing, China; b School of Life Science and Technology, China Pharmaceutical University, Nanjing, China

**Keywords:** Extraintestinal pathogenic *Escherichia coli*, elongation factor Tu, holo-transferrin, transferrin-binding protein, serum resistance

## Abstract

Extraintestinal pathogenic *Escherichia coli* (ExPEC) is a common anthropozoonotic pathogen that causes systemic infections. To establish infection, ExPEC must utilize essential nutrients including iron from the host. Transferrin is an important iron source for multiple bacteria. However, the mechanism by which ExPEC utilizes transferrin remains unclear. In this study, we found that iron-saturated holo-transferrin rather than iron-free apo-transferrin promoted the vitality of ExPEC in heat-inactivated human serum. The multifunctional protein Elongation factor Tu (EFTu) worked as a holo-transferrin binding protein. EFTu not only bound holo-transferrin rather than apo-transferrin but also released transferrin-related iron, with all domains of EFTu involved in holo-transferrin binding and iron release events. We also identified the surface location of EFTu on ExPEC. Overexpression of EFTu on the surface of nonpathogenic *E. coli* not only promoted the binding of bacteria to holo-transferrin but also facilitated the uptake of transferrin-related iron. More importantly, it significantly enhanced the survival of *E. coli* in heat-inactivated human serum, which was positively correlated with holo-transferrin but not apo-transferrin. Our research revealed a novel function of EFTu in binding holo-transferrin to promote iron uptake by bacteria, suggesting that EFTu was a potential virulence factor of ExPEC. In addition, our study provided research avenues into the iron acquisition and pathogenicity mechanisms of ExPEC.

## Introduction

Extraintestinal pathogenic *Escherichia coli* (ExPEC) is an important anthropozoonotic pathogen that causes infections in non-intestinal sites of humans and animals such as cerebrospinal fluid, the urinary tract, and even the bloodstream [1–3]. In addition, ExPEC can contaminate the food chain through poultry and pork products, imposing a serious threat to public health [4,5]. ExPEC possesses an array of enhanced virulence factors to establish infection in the hostile and nutrient-deficient host environment [6]. For example, the outer membrane protein A (OmpA) of ExPEC contributes to its ability to survive in serum and invade blood–brain endothelial cells [7,8]. The type I pilus adhesin of ExPEC helps bacteria to colonize and invade the bladder epithelium [9]. ExPEC *de novo* nucleotide biosynthetic pathways allow bacteria to meet their metabolic requirements within host blood and promote bacterial growth in serum [10].

Elongation factor Tu (EFTu) is a conserved GTPase in the cytoplasm that is crucial for protein biosynthesis [11]. It additionally plays a role in virulence-associated phenotypes as a surface protein of bacteria. EFTu in some bacteria has the capability to bind to host proteins. For example, the combination of EFTu and complement inhibitors, such as factor H, FHL-1, and plasminogen, enhances the ability of bacteria to escape from complement attack [12,13]. EFTu also binds to extracellular matrix proteins, including collagen, fibronectin, and laminin, which promotes the adhesion or invasion of bacteria to host cells [14–16]. There have been some reports on the surface location of EFTu on *E. coli* [17,18]. However, there remain few studies on the function of *E. coli* EFTu in binding to host proteins.

Transferrin is the major iron (Fe^3+^) carrier protein in human serum. There are three forms of transferrin in the host: iron-saturated holo-transferrin (with two molecules of iron), partially iron-saturated transferrin (with one molecule of iron), and iron-free apo-transferrin. Transferrin not only maintains the iron-restricted environment in the host by binding free iron, but also plays a role in transporting iron to host cells by recognizing transferrin receptors on the cell surface [19,20]. Some bacterial pathogens can utilize transferrin as a source of iron and have evolved transferrin-binding proteins to recruit holo-transferrin to their surfaces. For example, *Neisseria meningitidis* and *Neisseria gonorrhoeae* utilize the specialized transferrin-binding proteins TbpA and TbpB to recruit transferrin [21,22]. Bacteria that do not have specialized transferrin-binding proteins, such as *Mycobacterium tuberculosis*, *Staphylococcus aureus*, and *Staphylococcus epidermidis*, utilize surface-located GAPDH to bind transferrin [23–25]. Although studies have shown that enteropathogenic *E. coli* utilizes OmpA and OmpC to bind transferrin, there are few studies on transferrin-binding proteins of ExPEC [26]. Furthermore, whether ExPEC directly utilizes transferrin-related iron and the mechanism of iron uptake after binding to transferrin remains unknown.

Preliminary research in our laboratory revealed that ExPEC strain RS218 could recruit holo-transferrin to the bacterial surface. It was reported that the surface protein EFTu of *M. tuberculosis* is a holo-transferrin binding protein [23]. Considering the diverse functional roles and conserved genetic evolution of EFTu, we selected the protein for further research. This study focused on the interaction between EFTu and holo-transferrin to reveal the role of EFTu in the processes of *E. coli* binding to holo-transferrin, iron uptake, and serum survival.

## Materials and methods

### Strains, plasmids, and growth conditions

Bacterial strains and plasmids used in this study are listed in Table 1. RS218 (O18:H7:K1) is an ExPEC strain isolated from the cerebrospinal fluid of a human neonate [27]. *E. coli* DH5α was used as a host for plasmid cloning, and BL21 was used as a host for plasmid expression. Bacteria were cultured in Luria-Broth (LB) medium, solid LB medium, or M9 medium. M9 medium was prepared as described previously [3]. Briefly, 200 mL of 5 × M9 minimal salts (Sangon Biotech, Catalog No. A507024), 2 mL of 1 M MgSO_4_, 20 mL of 20% glucose, and 1 mL of 0.1 M CaCl_2_ was diluted to 1 L with double-distilled water and sterilized through a 0.22-μm filtration membrane. Antibiotics (kanamycin, 50 μg/mL; ampicillin, 100 μg/mL) were added to medium for culturing plasmid-carrying *E. coli* strains.

**Table 1.** Bacterial strains and plasmids used in this study

Names	Notable characteristic(s)^a^	Source or references
Bacteria		
DH5α	Host for plasmid cloning	Purchased from Vazyme
BL21 (DE3)	Host for recombinant protein expression	Purchased from Vazyme
RS218	ExPEC strain isolated from the cerebrospinal fluid in neonates	Sun et al., 2021
BL21-pEImC	BL21 (DE3) containing pEImC	This study
BL21-pEImC-EFTu	BL21 (DE3) containing pEImC:*eftu*	This study
BL21-pEImC-ΔTu1	BL21 (DE3) containing pEImC:Δtu1	This study
BL21-pEImC-ΔTu2	BL21 (DE3) containing pEImC:Δtu2	This study
BL21-pEImC-ΔTu3	BL21 (DE3) containing pEImC:Δtu3	This study
plasmids		
pET-32a	Prokaryotic recombinant protein expression plasmid, Amp^r^	Purchased from Takara
pET-28a	Prokaryotic recombinant protein expression plasmid, Kan^r^	Purchased from Takara
pET-28a:*eftu*	pET-28a containing *eftu*, Kan^r^	This study
pET-28a:Δtu1	pET-28a containing *eftu* with domain 1 deletion, Kan^r^	This study
pET-28a:Δtu2	pET-28a containing *eftu* with domain 2 deletion, Kan^r^	This study
pET-28a:Δtu3	pET-28a containing *eftu* with domain 3 deletion, Kan^r^	This study
pEImC	Surface display plasmid, Kan^r^	This study
pEImC:*eftu*	pEImC containing *eftu*, Kan^r^	This study
pEImC:Δtu1	pEImC containing *eftu* with domain 1 deletion, Kan^r^	This study
pEImC:Δtu2	pEImC containing *eftu* with domain 2 deletion, Kan^r^	This study
pEImC:Δtu3	pEImC containing *eftu* with domain 3 deletion, Kan^r^	This study

^a^Kan^r^, kanamycin resistance, Amp^r^, ampicillin resistance.

### Preparation of transferrin-deficient serum

Normal human serum (Sigma-Aldrich, Catalog No. H4522) was obtained from human male AB plasma. Heat-inactivated human serum (HIHS) was prepared by incubating normal serum for 30 min at 56°C Transferrin-deficient HIHS was prepared as described previously with modifications [27,28]. Briefly, 100 μg rabbit IgG (Sigma-Aldrich, Catalog No. 12–370) or rabbit anti-transferrin antibody (Abcam, Catalog No. ab66952) was incubated with Protein A/G magnetic agarose beads (Thermo Scientific, Catalog No. 78,609) for 2 h at 4°C Excess antibodies were washed away using PBS before incubating beads with HIHS for 2 h at 4°C Protein A/G beads were then removed, and HIHS was collected. Anti-transferrin antibody (1:150,000) and HRP-conjugated anti-rabbit IgG (1:5000) were used to detect iron carrying transferrin signals in transferrin-deficient HIHS and control HIHS after the above treatment using an ELISA. The ELISA assays were repeated six times.

### Serum survival and medium growth assays of ExPEC

Serum survival assays were performed as described previously with some modifications [29–31]. ExPEC strain RS218 was cultured in LB medium to the logarithmic growth phase. Fresh bacteria were washed twice with M9 medium. Then, 1 × 10^7^ CFUs of bacteria were incubated with 20% transferrin-deficient HIHS, 20% transferrin-deficient HIHS supplemented with 200 μg/mL human holo-transferrin (Sigma, Catalog No. T0665) or 200 μg/mL human apo-transferrin (Sigma, Catalog No. T2036), or 20% control HIHS in M9 medium at 37°C, respectively. After 3 h of incubation, bacteria were counted by plate counting. Data were calculated as the ratio of the number of bacteria recovered from serum to the original number of bacteria. To identify the effect of holo-transferrin and apo-transferrin in HIHS on the viability of ExPEC, the growth ability of ExPEC RS218 in M9 medium and M9 medium supplemented with holo-transferrin or apo-transferrin was also tested as described above. These experiments were repeated six times independently.

### Expression of recombinant proteins and antibody preparation

Primers used in this study are listed in Table 2. The *eftu* gene and its domain deletion mutants (Δtu1, Δtu2, and Δtu3) were recombined into pET-28a plasmid. Recombinant proteins were expressed and purified as previously described [27].

**Table 2.** Primers used in this study

Names	Oligonucleotide sequence (5´–3´)^a^	Products
EFTu-F	TGGACAGCAAATGGGTCGCGGATCCATGTCTAAAGAAAAGTTTGA	*eftu*
EFTu-R	GAGTGCGGCCGCAAGCTTGTCGACGTTAGCTCAGAACTTTTGCTA	
ΔTu1-F	ATGGGTCGCGGATCCGAATTCATGCCAGAGCGTGCGATTGACAAG	Δtu1
ΔTu1-R	GTGGTGGTGGTGGTGCTCGAGTTAGCCCAGAACTTTAGCAAC	
ΔTu2-F1	ATGGGTCGCGGATCCGAATTCGTGTCTAAAGAAAAATTTGAACGTACAA	Δtu2-1
ΔTu2-R1	TTACGACCGGAGATGGAGAATACG	
ΔTu2-F2	TTCTCCATCTCCGGTCGTAAGCCGGGCACCATCAAG	Δtu2-2
ΔTu2-R2	GTGGTGGTGGTGGTGCTCGAGTTAGCCCAGAACTTTAGCAACAAC	
ΔTu3-F	ATGGGTCGCGGATCCGAATTCGTGTCTAAAGAAAAATTTGAAC	Δtu3
ΔTu3-R	GTGGTGGTGGTGGTGCTCGAGTTAGCCCGGCTTAGCCAGTACCTGAC	
EFTu-pEImC-F	GGTTCTGGATCCGAATTCGAGCTCATGTCTAAAGAAAAATTTGAAC	eftu-pEImC
EFTu-pEImC-R	TGGTGGTGGTGGTGGTGCTCGAGTTTAGCCCAGAACTTTAGCAAC	
ΔTu1-pEImC-F	GGTTCTGGATCCGAATTCGAGCTCATGCCAGAGCGTGCGATTGACAAG	Δtu1-pEImC
ΔTu1-pEImC-R	TGGTGGTGGTGGTGGTGCTCGAGTTTAGCCCAGAACTTTAGCAAC	
ΔTu2-pEImC-F	GGTTCTGGATCCGAATTCGAGCTCGTGTCTAAAGAAAAATTTGAACGTACAA	Δtu2-pEImC
ΔTu2-pEImC-R	TGGTGGTGGTGGTGGTGCTCGAGTTTAGCCCAGAACTTTAGCAAC	
ΔTu3-pEImC-F	GGTTCTGGATCCGAATTCGAGCTCGTGTCTAAAGAAAAATTTGAAC	Δtu3-pEImC
ΔTu3-pEImC-R	TGGTGGTGGTGGTGGTGCTCGAGTTTAGCCCGGCTTAGCCAGTACCTGAC	

^a^Underlined sequences correspond to restriction enzyme recognition sites.

GGATCC, BamH I; GTCGAC, Sal I; GAATTC, EcoR I; CTCGAG, Xho I; GAGCTC, Sac I.

Rabbit anti-EFTu antibody was prepared by Shanghai Willget Biotechnology Co., Ltd. The peptide used as the immunogen was SKEKFERTKPHVN. Western blotting was performed as described previously to determine the anti-EFTu antibody [32].

### Determination of EFTu surface location of ExPEC

To determine the surface location of EFTu on ExPEC RS218, immunofluorescence assays, colony blotting, and Western blotting were performed as previously described [27].

For immunofluorescence assays, ExPEC strain RS218 was cultured in LB medium to the logarithmic growth phase. Fresh bacteria were collected by centrifugation, washed with PBS, and fixed in paraformaldehyde at 4°C for 1 h. After washing and blocking, bacteria were incubated with a mixture of mouse anti-RS218 antiserum (1:500) and one of three antibodies: rabbit anti-EFTu (1:1000), rabbit anti-OmpA (1:1000, outer membrane protein control), and rabbit anti-LexA (1:2000, Abcam, Catalog No. ab174384). The LexA is a cytoplasmic protein of *E. coli*. To stain cytoplasmic proteins, fixed RS218 cells were washed and then incubated with GTE buffer [50 mM glucose, 25 mM Tris (pH 8), 10 mM EDTA] overnight at 4°C. To permeabilize cell membranes, bacteria were incubated with lysozyme (2 mg/mL) and EDTA (5 mM) for 45 minutes at room temperature [33]. After washing and blocking, permeabilized bacteria were incubated with a mixture of mouse anti-RS218 antiserum and rabbit anti-LexA antibody. After washing, all bacteria were incubated with the mixture of FITC-conjugated goat anti-mouse IgG (1:1000, Abcam, Catalog No. ab6785) and TRITC-conjugated goat anti-rabbit IgG (1:1000, Abcam, Catalog No. ab6718). Bacteria were highlighted by staining them with DAPI (1 μg/mL, Sigma, Catalog No. MBD0015) for 10 min at room temperature. Bacteria were washed four times with PBS by centrifugation. Then bacteria cells were resuspended in PBS and placed on a coverslip. The fluorescent signals were observed under a fluorescence microscope.

For colony blotting assay, RS218 strain was spread on solid LB medium and cultured overnight. After covering the colonies with nitrocellulose (NC) membranes for 5 min, the NC membranes were air-dried for an additional 30 min. After washing and blocking, the NC membranes were incubated with antibodies against EFTu (1:1000), OmpA (1:1000), and LexA (1:2000), respectively. Detections were carried out by hybridization with HRP-conjugated anti-rabbit IgG (1:5000).

For Western blotting, the surface and cytoplasmic proteins of ExPEC strain RS218 were isolated as described previously [27,34]. The whole bacteria proteins, surface proteins, and cytoplasmic proteins of ExPEC were separated by SDS-PAGE, and then transferred to PVDF membranes. The PVDF membranes were blocked with BSA, then incubated with anti-EFTu, anti-OmpA, and anti-LexA antibodies, respectively. Detections were carried out by hybridization with HRP-conjugated anti-rabbit IgG.

The above experiments were independently repeated three times.

### Detection of the interaction between EFTu and transferrin

Far-Western blotting assays were performed as previously described with few modifications [23]. Briefly, holo-transferrin, recombinant EFTu (rEFTu), and recombinant His (rHis, obtained from BL21-strain carrying pET-32a) were denatured and subjected to SDS-PAGE. Proteins were transferred to a PVDF membrane. Far-Western blotting was carried out by incubating the PVDF membrane with rEFTu (10 μg/mL), anti-EFTu antibody (1:1000), and HRP-conjugated goat anti-rabbit IgG (1:5000). This assay was independently repeated three times.

For ELISA plate-binding assay, rEFTu (0.01, 0.1, 0.2, and .5 μM), ΔTu1 (0.2 μM), ΔTu2 (0.2 μM), ΔTu3 (0.2 μM), and rHis were coated on wells of a 96-well ELISA plate overnight at 4°C Wells were blocked with 2% BSA in PBST (PBS with 0.05% Tween-20), followed by the incubation with 0.2 μM desthiobiotin-conjugated holo-transferrin (desthiobiotinylated holo-transferrin). After washing, wells were incubated with HRP-streptavidin (1:5000, Invitrogen, Catalog No. 43–4323) for 2 h at 4°C Wells not coated with proteins were used as controls, and values were subtracted from those of test samples. The interaction between EFTu and desthiobiotin-conjugated apo-transferrin (desthiobiotinylated apo-transferrin) was also detected as described above. Desthiobiotinylated holo-transferrin and apo-transferrin was prepared by labeling holo-transferrin or apo-transferrin with EZ-Link sulfo-NHS-LC desthiobiotin (Thermo Fisher Scientific, Catalog No. 16138) according to the manufacturer’s instructions. This assay was independently repeated four times.

For protein-binding inhibition assays, rEFTu (0, 0.1, 0.2, and 0.5 μM) was incubated with fluorescein-conjugated holo-transferrin (0.2 μM, Life, Catalog No. T2871) for 2 h at 4°C followed by incubation with ExPEC strain RS218 for 2 h at 4°C. After washing with PBS, the fluorescence intensity of 1.0 × 10^7^ bacteria was detected for fluorescein-holo-transferrin at an excitation wavelength of 485 nm and emission wavelength of 535 nm. RS218 strain incubated with fluorescein-holo-transferrin alone was used as a positive control. The background value for bacteria incubated without fluorescein-holo-transferrin was subtracted from the fluorescence intensity of samples. Data were calculated as fold-change relative to the positive group. This assay was independently repeated five times.

### Detection of holo-transferrin conversion by EFTu

Desthiobiotinylated holo-transferrin (0.1 μM) was incubated with rEFTu (0.1, 0.2, 0.5, 1 μM), ΔTu1 (1 μM), ΔTu2 (1 μM), and ΔTu3 (1 μM), respectively, at 37°C in a total volume of 200 μL. Desthiobiotinylated holo-transferrin incubated without proteins served as a positive control. After 2 h of incubation, the protein mixtures were diluted and coated on the ELISA plate. OD_450_ values of transferrin signals and desthiobiotin signals were measured using anti-transferrin antibody (1:150,000) and HRP-streptavidin (1:5000), respectively. Wells not coated with proteins were used as a blank control, and OD_450_ values were subtracted from those of samples. Relative OD_450_ values were calculated as the ratio of the OD_450_ value of iron-carrying transferrin signals to that of total transferrin signals. This assay was independently repeated five times.

### Surface display plasmid construction and verification

To express a heterologous protein on the surface of *E. coli*, a surface display plasmid was constructed as described previously with some modifications [35–37]. Nucleotide sequences encoding the *N*-terminal domain of ice nucleoprotein (InaZN, Accession No. PBP57058), the GGGGS linker, mCherry (GenBank: MK753226.1), and another GGGGS linker were fused and recombined into pET-28a using *Nco* I and *Bam*H I. InaZN functioned as an anchoring motif and mCherry functioned as a reporter. All genes were optimized based on the codon usage bias of *E. coli*. The constructed vector was termed pEImC. *eftu*, Δtu1, Δtu2, and Δtu3 were recombined into the pEImC plasmid and transformed into *E. coli* BL21, respectively. *E. coli* BL21 containing pEImC, pEImC:*eftu*, pEImC:Δtu1, pEImC:Δtu2, or pEImC:Δtu3 plasmid was termed BL21-pEImC, BL21-pEImC-EFTu, BL21-pEImC-ΔTu1, BL21-pEImC-ΔTu2, and BL21-pEImC-ΔTu3, respectively. These above five BL21 strains containing a series of pEImC plasmids were cultured in LB broth medium at 37°C until the value of OD_600_ reached 0.3. Fusion protein expressions were induced by adding 0.5 mM isopropyl-β-D-thiogalactopyranoside for another 4 h.

Expression of fused proteins was detected by separating induced whole bacteria using SDS-PAGE and observing mCherry fluorescence signals under a fluorescence microscope. The fluorescence intensity of bacteria (1 × 10^7^ CFUs) at an excitation wavelength of 579 nm and an emission wavelength of 624 nm was measured to determine mCherry expression levels. Background values of uninduced bacteria were subtracted from the fluorescence intensity of samples. Surface expression of mCherry on five BL21 strains carrying pEImC a series of plasmids was also measured using a whole-cell ELISA assay with rabbit anti-mCherry antibody (Abcam, Catalog No. ab213511) as described previously [27]. Surface expression of EFTu in BL21-pEImC-EFTu and BL21-pEImC was measured using a whole-cell ELISA assay with anti-EFTu antibody. In addition, surface proteins and cytoplasmic proteins of BL21-pEImC-EFTu and BL21-pEImC were extracted as described previously and subjected to Western blotting [27]. Anti-EFTu and anti-OmpA antibodies were used to detect blotting signals in surface fractions, and anti-EFTu and anti-LexA antibodies were used to detect blotting signals in cytoplasmic fractions. Gray intensities of protein bands were analyzed by Image J software. These above assays were independently repeated five or six times.

### Holo-Transferrin binding assays of BL21 strains carrying a series of pEimc plasmids

These five induced BL21 strains were washed three times with PBS. After blocking with 2% BSA, bacteria (2.0 × 10^8^ CFUs) from each strain were incubated with 20 μg fluorescein-holo-transferrin at 4°C for 1 h, respectively. Bacteria incubated with holo-transferrin were used as a background control. The fluorescence intensity of fluorescein-holo-transferrin was measured as described for recombinant protein inhibition assays. Values for the background control were subtracted from those of samples. This assay was independently repeated five times.

### Iron uptake determination of BL21 strains carrying a series of pEimc plasmids

Calcein-AM fluorescence-quenching assays were performed to detect the transferrin-iron uptake of bacteria as described previously with some modifications [23,38]. Briefly, the five BL21 strains carrying a series of pEImC plasmids were induced in LB broth medium and washed three times with M9 medium. Bacteria were incubated with calcein-AM (1 μM, Sigma, Catalog No. C1359) for 150 min at 37°C in M9 medium and then washed three times before incubating with 10 μg/mL holo-transferrin in M9 medium for 6 h. Bacteria incubated without holo-transferrin were used as a negative control. After washing with M9 medium, the fluorescence intensity of calcein in the bacteria (1 × 10^7^ CFUs) was measured at excitation 490 nm and emission 538 nm. This assay was independently repeated five times.

To detect the conversion of holo-transferrin, 1 × 10^8^ cells of the five BL21 strains carrying a series of pEImC plasmids were washed with M9 medium and incubated with 10 μg/mL desthiobiotinylated holo-transferrin at 37°C. After 6 h of incubation, bacteria were placed on ice for 10 min. Culture supernatants were collected by centrifugation at 10,000 × *g* for 10 min and coated on an ELISA plate. Iron-carrying transferrin and total transferrin signals were detected using an anti-transferrin antibody (1:150,000) and HRP-streptavidin (1:5000), respectively. Desthiobiotinylated holo-transferrin that did not interact with bacteria was used as a positive control. The value of the M9 medium was subtracted from values of all samples. Relative values of OD_450_ were calculated as the ratio of iron-carrying signals to total transferrin signals. This assay was independently repeated five times.

### Serum survival and medium growth assays of BL21 strains carrying a series of pEimc plasmids

For the five BL21 strains carrying a series of pEImC plasmids, bacteria were washed with M9 medium and then incubated with 20% transferrin-deficient HIHS, 20% transferrin-deficient HIHS supplemented with 200 μg/mL holo-transferrin or 200 μg/mL apo-transferrin, or 20% control HIHS in M9 medium at 37°C for 3 h. Survival ratios were calculated as described above. To determine the sensitivity of different BL21 strains carrying a series of pEImC plasmids to M9 medium, the growth ratios of bacteria in M9 medium were also determined. In addition, growth abilities of the five BL21 strains carrying a series of pEImC plasmids in M9 medium supplemented with holo-transferrin and apo-transferrin were also determined. This assay was independently repeated five times.

### Statistical analysis

All experiments were repeated at least three times. GraphPad Prism version 8.0 was used to analyze and plot data. Data are represented as means with standard errors. Statistical analyses were assessed using unpaired *t* test and one-way ANOVA. The significant difference was accepted as *P* < 0.05.

## Results

### Holo-Transferrin promotes serum viability of ExPEC

**Figure 1.**
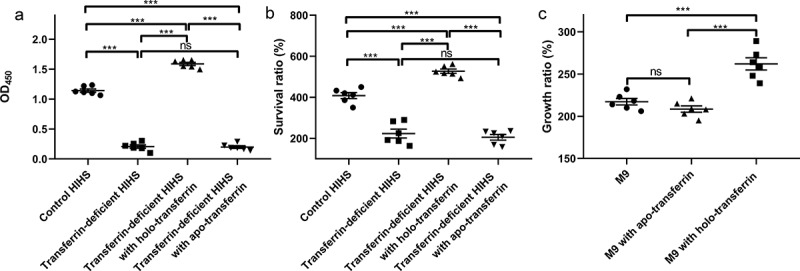
Holo-Transferrin enhances the ability of ExPEC to survive in serum. (a) the transferrin-deficient HIHS, control HIHS, transferrin-deficient HIHS supplemented with holo-transferrin, or apo-transferrin were prepared, then their iron-carrying transferrin signals were detected using ELISA with anti-transferrin antibody. (b) ExPEC strain RS218 was incubated with transferrin-deficient HIHS, control HIHS, transferrin-deficient HIHS supplemented with holo-transferrin or apo-transferrin for 3 h and recovered bacteria were counted. Survival ratios were calculated as the ratio of the number of bacteria recovered from HIHS to the number of original bacteria (1.0 × 10^7^ CFUs). (C) ExPEC strain RS218 was incubated in M9 medium, apo-transferrin supplemented M9 medium, or holo-transferrin supplemented M9 medium for 3 h. Growth abilities were calculated as the ratio of the number of bacteria recovered from medium to the number of original bacteria (1.0 × 10^7^ CFUs). Data are represented mean ± standard error. Statistically differences were determined using unpaired *t-*test. **P <0.005; ***P <0.001; ns, not significant.

As an important iron source in serum, transferrin can be utilized by some bacteria. To explore the effect of transferrin on ExPEC, we prepared transferrin-deficient HIHS and then evaluated the ability of ExPEC RS218 to tolerate serum. As shown in Figure 1A, the levels of iron-carrying transferrin in transferrin-deficient HIHS were significantly lower than those in the control HIHS and the transferrin-deficient HIHS supplemented with holo-transferrin, indicating the successful preparation of transferrin-deficient HIHS (*P* < 0.001). Transferrin levels in the control HIHS and transferrin-deficient HIHS supplemented with apo-transferrin were not significantly different (*P* > 0.05, Figure 1A) because the anti-transferrin antibody is not suitable for ELISA detection of apo-transferrin (Fig. S1). Compared with the control group, the survival ratio of ExPEC RS218 in transferrin-deficient HIHS was significantly decreased, while the survival ratio in holo-transferrin supplemented HIHS was significantly increased (*P* < 0.001, Figure 1B). The survival ratio of ExPEC in apo-transferrin supplemented HIHS was not significantly different from that in transferrin-deficient HIHS, which excluded the influence of apo-transferrin on the results (*P* > 0.05, Figure 1B). The composition of serum is complex and may contain other factors that affect bacterial serum survival ability. To explore the relationship between ExPEC serum tolerance and transferrin, we also determined the growth ratio of ExPEC strain RS218 in M9 medium. The addition of apo-transferrin did not affect the growth of ExPEC RS218 in M9 medium, while the addition of holo-transferrin resulted in a significant increase in the growth ratio of ExPEC (*P* < 0.01Figure 1C), indicating that the iron carried by transferrin, not transferrin itself, was the factor affecting the growth of bacteria. These results indicated that the holo-transferrin in HIHS conferred the tolerance of ExPEC to serum.

### EFTu is located on the surface of ExPEC

**Figure 2.**
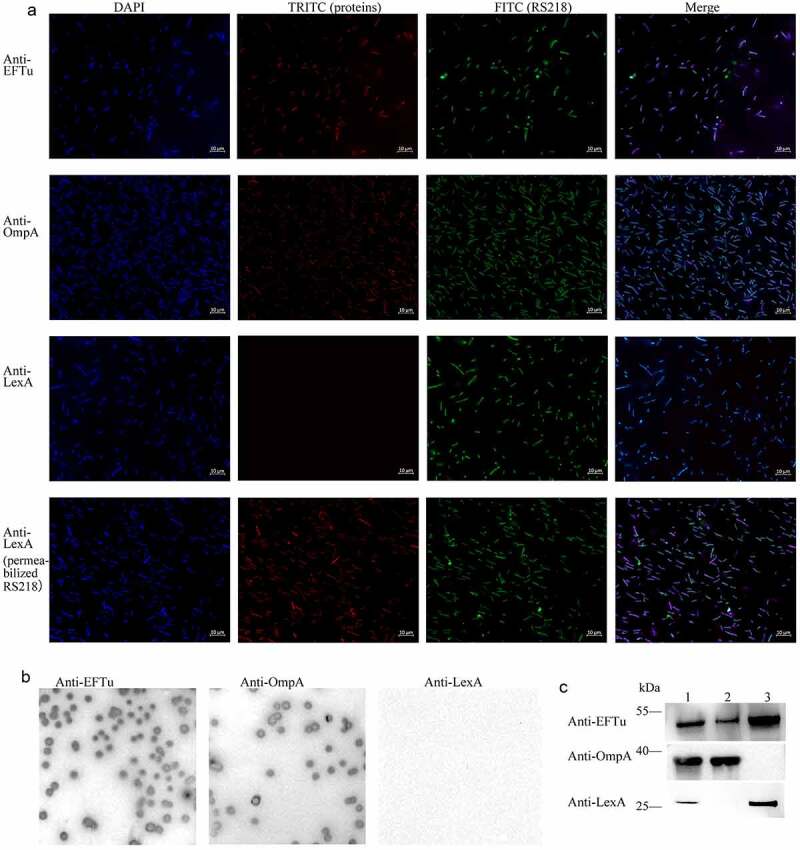
Eftu is located on the surface of ExPEC. (a) Intact ExPEC RS218 bacteria were incubated with a mixture of mouse anti-RS218 antiserum and rabbit anti-EFTu, rabbit anti-OmpA, or rabbit anti-LexA antibody. Permeabilized RS218 cells were incubated with a mixture of mouse anti-RS218 antiserum and rabbit anti-LexA antibody. Cells were then incubated with a mixture of FITC-conjugated anti-mouse IgG and TRITC-conjugated anti-rabbit IgG. to highlight the bacteria, they were stained with DAPI. Fluorescence signals of DAPI, FITC, and TRITC were detected using a fluorescence microscope. Bars, 10 μm. (b) Colonies of ExPEC strain RS218 were covered with NC membrane followed by incubation with antibody against EFTu, OmpA, or LexA. (c) Whole bacterial, surface, and cytoplasmic proteins of ExPEC were subjected to Western blotting and hybridized to antibody against EFTu, OmpA, or LexA. Lane 1, the whole bacterial fractions of RS218 strain; lane 2, the surface proteins; lane 3, the cytoplasmic fractions of RS218.

In some bacteria, such as *Pseudomonas aeruginosa*, *Lactobacillus johnsonii*, and *Streptococcus gordonii*, EFTu is not only located in the cytoplasm, but also on the cell surface [13,37,38]. To investigate whether EFTu was the surface protein of ExPEC, we performed immunofluorescence and immunoblotting. Co-localization of the FITC signal representing RS218 and the TRITC signal representing OmpA and EFTu indicated that EFTu was localized on the surface of ExPEC RS218. The negative TRITC signal representing LexA of intact bacteria and the positive signal of membrane-damaged bacteria indicated that cytoplasmic proteins were not detectable on the surface of intact bacteria (Figure 2A). Positive signals for OmpA and EFTu as well as the negative result for LexA on the surface of RS218 colonies indicated the surface localization of EFTu (Figure 2B). As Figure 2C shown, EFTu bands (approximately 55 kDa) were detected in RS218 whole bacteria fractions, surface proteins, and cytoplasmic proteins. OmpA bands (approximately 40 kDa) were detected in whole bacterial proteins and surface proteins, but not in cytoplasmic fractions, indicating that the extracted cytoplasmic proteins did not contain surface fractions. LexA bands (approximately 25 kDa) were detected in whole bacterial proteins and cytoplasmic proteins, but not in surface fractions, excluding the contamination of the membrane proteins in the cytoplasmic fractions. These above results indicated that EFTu is not only a cytoplasmic protein, but also localizes to the outer membrane of ExPEC RS218.

### Eftu binds to holo-transferrin and releases transferrin-related iron

**Figure 3.**
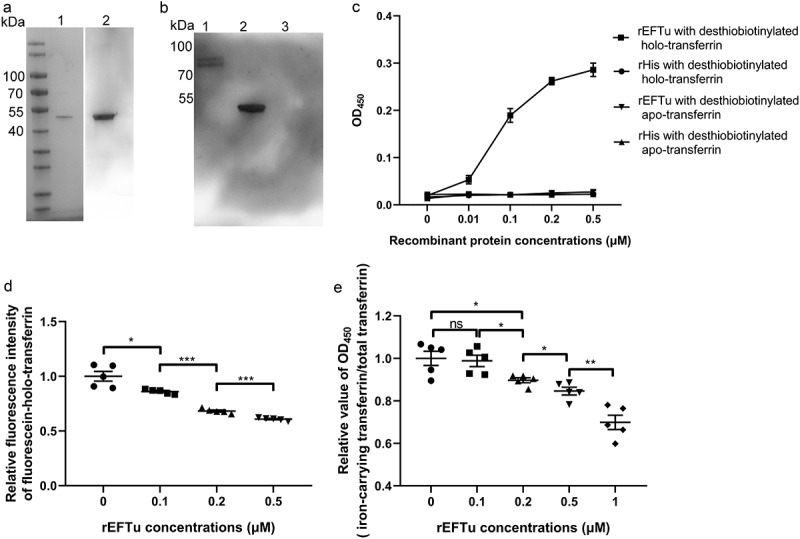
Eftu interacts with holo-transferrin. (a) rEftu was subjected to SDS-PAGE and stained with Coomassie G-250 (lane 1). the rEftu on PVDF membrane was hybridized to anti-EFTu antibody (lane 2). (b) Far-western blotting analysis of holo-transferrin with rEftu. Holo-transferrin (lane 1), rEftu (lane 2), and rHis (negative control, lane 3) were loaded on the PVDF membrane and then incubated with rEftu. the interactions were detected using anti-EFTu antibody. (c) Binding of rEftu and rHis to desthiobiotinylated holo-transferrin or desthiobiotinylated apo-transferrin was detected using ELISA plates coated with different concentrations of recombinant proteins. OD_450_ values were obtained by incubating with HRP-streptavidin. (d) Different concentrations of rEftu and fluorescein-holo-transferrin were pre-incubated before incubating with ExPEC RS218. Fluorescein-holo-transferrin directly incubated with ExPEC was used as a positive control. the fluorescence intensity of fluorescein-holo-transferrin bound by the bacteria was detected. Relative fluorescence intensity was calculated as the ratio of the treatment group to the positive control. (e) Different concentrations of rEftu incubated with desthiobiotinylated holo-transferrin. Protein mixtures were coated on the ELISA plate. Iron-carrying transferrin and total transferrin signals were detected using anti-transferrin antibody and HRP-streptavidin, respectively. Desthiobiotinylated holo-transferrin that incubated without rEftu served as a control group. the relative value of OD_450_ was calculated as the ratio of the OD_450_ value of iron-carrying transferrin signals to that total transferrin signals. Data are represented mean ± standard error. Statistical differences were determined using unpaired *t* test. ns, not significant; *P <0.05; **P <0.005.

To explore the function of EFTu, we recombinantly expressed EFTu (Figure 3A) and detected the interaction between EFTu and holo-transferrin. In the far-Western blotting assay, the EFTu signal was detected in the holo-transferrin lane on the PVDF membrane, while the EFTu signal was not detected in the rHis lane, indicating that rEFTu combined with holo-transferrin (Figure 3B). The ELISA plate binding assay showed that the interaction between rEFTu and holo-transferrin was concentration-dependent (Figure 3C). In addition, the ELISA plate binding assay confirmed that there was no interaction between EFTu and apo-transferrin (Figure 3C). The protein-binding inhibition assay showed that pre-incubation of rEFTu and fluorescein-holo-transferrin resulted in a significant reduction of fluorescein-holo-transferrin bound to ExPEC RS218 in an EFTu dose-dependent manner (*P* < 0.05, Figure 3D).

According to the manufacturer’s instructions, the anti-transferrin antibody reacted with partially iron-saturated transferrin. In preliminary studies, it was found that this antibody is also suitable for detecting holo-transferrin in ELISA, but not for detecting apo-transferrin (Fig. S1). The HRP-streptavidin reacts with desthiobiotin on all forms of transferrin (total transferrin), including holo-transferrin, partially saturated transferrin, and apo-transferrin. When desthiobiotin-labeled holo-transferrin released iron and converted into apo-transferrin, the transferrin signal intensity decreased while the desthiobiotin signal intensity remained unchanged. Therefore, the ratio of transferrin signal intensity to desthiobiotin signal intensity (iron-carrying transferrin/total transferrin) can reflect the production of apo-transferrin. To determine the ability of EFTu to release transferrin-related iron, the production of apo-transferrin was detected. The incubation of rEFTu with holo-transferrin resulted in a significant decrease relative OD_450_ values (iron-carrying transferrin/total transferrin) in an EFTu dose-dependent manner (*P* < 0.05, Figure 3E), implying the release of transferrin-related iron release of transferrin-related iron.

The above results indicated that EFTu of ExPEC not only specifically bound holo-transferrin and not apo-transferrin, but also released the transferrin-related iron.

### Determination of holo-transferrin-binding domain and iron-releasing domain of EFTu

**Figure 4.**
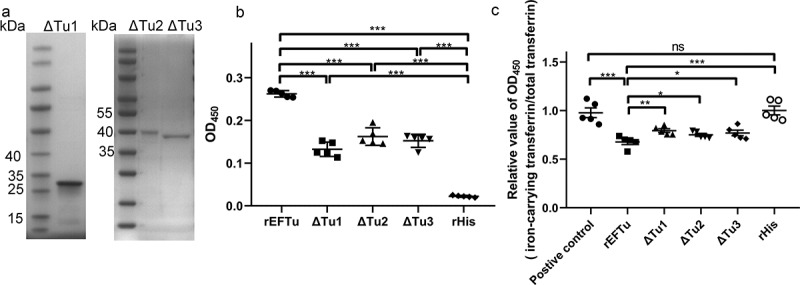
All domains of EFTu contribute to binding holo-transferrin and transferrin-related iron release. (a) δtu1, δtu2, and δtu3 were subjected to SDS-PAGE and stained with Coomassie G-250. (b) Binding of δtu1, δtu2, and δtu3 to desthiobiotinylated holo-transferrin was detected using ELISA plates coated with recombinant proteins. rHis was used as a negative control. OD_450_ was obtained by incubating with HRP-conjugated streptavidin. (c) δtu1, δtu2, and δtu3 were incubated with desthiobiotinylated holo-transferrin. Protein mixtures were coated on the ELISA plate. Iron-carrying transferrin and total transferrin signals were detected using anti-transferrin antibody and HRP-streptavidin, respectively. the same dosage of desthiobiotinylated holo-transferrin was used as a positive control. the relative value of OD_450_ was calculated as the ratio of the OD_450_ value of iron-carrying transferrin signals to that total transferrin signals. Data are expressed as the mean ± standard error. Statistical differences were determined using unpaired *t* test. ns, not significant, *P <0.05; **P <0.005.

According to the prediction results of pfam (http://pfam.xfam.org/), EFTu contained three domains, namely the GTP_EFTU domain (amino acids 10–202), GTP_EFTU_D2 domain (amino acids 225–294), and GTP_EFTU_D3 domain (amino acids 298–393). In this study, the *N*-terminus of EFTu and the GTP_EFTU domain was regarded as Tu1. The GTP_EFTU_D2 domain was regarded as Tu2. And the GTP_EFTU_D3 domain and the C-terminus of EFTu was regarded as Tu3. To study the functions of the different domains, we expressed recombinant proteins with domain deletions (Figure 4A). ELISA plate binding assays showed that recombinant proteins with any of the domains deleted retained their holo-transferrin binding ability, although their holo-transferrin binding abilities were significantly reduced compared with that of the full-length rEFTu (*P* < 0.001, Figure 4B). Detection of transferrin-related iron release showed that relative OD_450_ values (iron-carrying transferrin/total transferrin) of ΔTu1, ΔTu2, and ΔTu3 were significantly reduced compared with those of the positive control, which indicated that transferrin-related iron was released (*P* < 0.01, Figure 4C). Compared with full-length EFTu, although the deletion of any one of the domains significantly weakened the ability of EFTu to release transferrin-related iron, none of these deletions abolished these functions of EFTu (*P* < 0.05). The above results indicated that each domain of EFTu was involved in the process of transferrin binding and transferrin-related iron release. And none of the three domains seems indispensable for the ability of EFTu to link holo-transferrin and release transferrin-related iron.

### The availability of surface display strains

**Figure 5.**
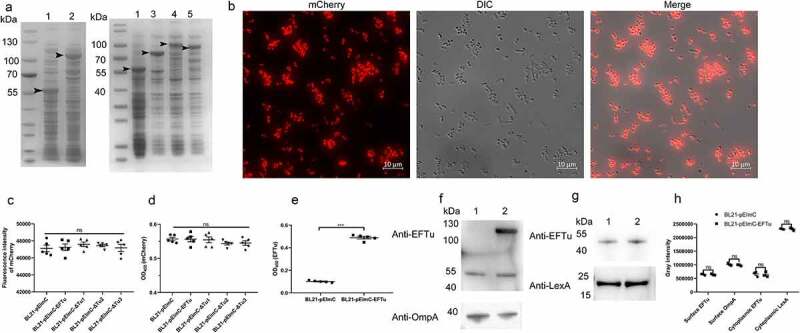
Availability of surface-expressed proteins in BL21 strains carrying a series of pEimc plasmids. (a) Whole bacterial proteins from induced BL21 strains carrying a series of pEimc plasmids were separated by SDS-PAGE and stained with Coomassie G-250. Lane 1, BL21-pEimc; lane 2, BL21-pEimc-EFTu; lane 3, BL21-pEimc-δtu1; lane 4, BL21-pEimc-δtu2; lane 5, BL21-pEimc-δtu3. Arrowheads indicate overexpression of fusion proteins. (b) Induced BL21-pEimc strain observed under a fluorescence microscope. Bars, 10 μm. (c) mCherry fluorescence intensities of the five induced BL21 strains carrying a series of pEimc plasmids were detected at an excitation of 579 nm and an emission of 624 nm. (d) Whole bacteria of the five induced BL21 strains carrying a series of pEimc plasmids were coated onto an ELISA plate. Expression levels of mCherry on the bacterial surface were detected using anti-mCherry antibody. (e) Whole bacteria of induced BL21-pEimc and BL21-pEimc-EFTu strains were coated onto an ELISA plate. the expression level of EFTu on the bacterial surface was detected using anti-EFTu antibody. (f) Surface fractions of induced BL21-pEimc (lane 1) and BL21-pEimc-EFTu (lane 2) strains were detected using Western blotting with anti-EFTu antibody and anti-OmpA antibody. (g) Cytoplasmic fractions of induced BL21-pEimc (lane 1) and BL21-pEimc-EFTu strain (lane 2) were detected using Western blotting with anti-EFTu and anti-LexA antibodies. (h) Gray intensities analysis of protein bands in panel F and G. Data are represented mean ± standard error. Statistical differences in panel C and D were determined using one-way ANOVA, and those in panel E and H were determined using unpaired *t-*test. ns, not significant; ***P <0.001.

SDS-PAGE of whole bacterial proteins from induced BL21-pEImC, BL21-pEImC-EFTu, BL21-pEImC-ΔTu1, BL21-pEImC-ΔTu2, and BL21-pEImC-ΔTu3 strains was shown in Figure 5A. Induced expression of a band of approximately 55 kDa in strain BL21-pEImC indicated that InaZN and mCherry were successfully fused and expressed. Similarly, overexpression bands in BL21-pEImC-EFTu, BL21-pEImC-ΔTu1, BL21-pEImC-ΔTu2, and BL21-pEImC-ΔTu3 indicated that InaZN, mCherry, and EFTu or its domain-deletion proteins were successfully fused and expressed. Fluorescence microscopy revealed strong fluorescence signals in the induced BL21-pEImC strain (Figure 5B). As shown in Figure 5C and 5D, there was no significant difference in the fluorescence intensity or the surface expression level of mCherry (*P* > 0.05) among the five BL21 strains carrying a series of pEImC plasmids, suggesting that there was no significant difference in the expression levels of recombinant protein among them. The surface expression level of EFTu on the BL21-pEImC-EFTu strain was significantly higher than that on the BL21-pEImC strain (*P* < 0.001, Figure 5E). In addition, surface proteins and cytoplasmic proteins of BL21-pEImC and BL21-pEImC-EFTu strains were extracted and detected using anti-EFTu antibody by Western blotting. As shown in Figure 5F, there were two bands reacting with the EFTu antibody among the surface proteins of the BL21-pEImC-EFTu strain, one of which was approximately 110 kDa, corresponding to expressed fusion EFTu, and the other approximately 50 kDa, corresponding to endogenous EFTu of BL21 strain. Detection of OmpA served as a reference for bacterial surface protein. Western blotting of cytoplasmic proteins of BL21-pEImC and BL21-pEImC-EFTu strains also detected bands reacting with EFTu antibody and LexA antibody (Figure 5G). Gray intensity analysis showed that expression levels of endogenous EFTu and OmpA on bacterial surface and EFTu and LexA in bacterial cytoplasm among BL21-pEImC and BL21-pEImC-EFTu were not significantly different (*P* > 0.05, Figure 5H), which indicated that surface overexpression of EFTu affected neither outer membrane protein nor cytoplasmic protein expression.

The above results indicated the successful construction and available applications of surface expression a series of pEImC vectors.

### Overexpressed EFTu promotes the ability of E. coli to bind holo-transferrin and uptake transferrin-related iron

**Figure 6.**
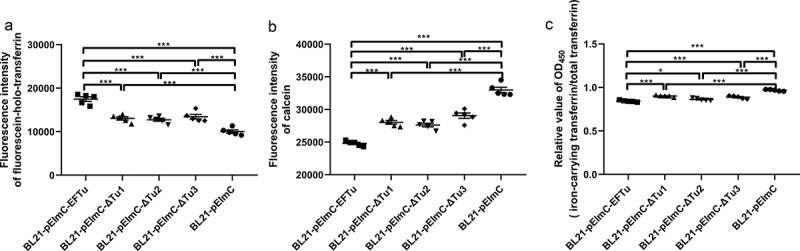
Overexpressed EFTu promotes the binding of holo-transferrin and iron uptake by *E. coli*. (a) BL21-pEimc, BL21-pEimc-EFTu, BL21-pEimc-δtu1, BL21-pEimc-δtu2, and BL21-pEimc-δtu3 strains were incubated with fluorescein-holo-transferrin, then the fluorescence intensity of fluorescein-holo-transferrin bound to bacteria was detected. (b) Five BL21 strains carrying a series of pEimc plasmids were stained with calcein-AM. Quenching of calcein fluorescence indicating iron uptake was measured in the presence of holo-transferrin at excitation 490 nm and emission 538 nm. (c) Five BL21 strains carrying a series of pEimc plasmids were incubated with desthiobiotinylated holo-transferrin in M9 medium. Protein in supernatants were coated on the ELISA plate. Iron-carrying transferrin and total transferrin signals were detected using anti-transferrin antibody and HRP-streptavidin, respectively. Relative values of OD_450_ were calculated as the ratio of the iron-carrying transferrin signal to total transferrin signal. Data are expressed as the mean ± standard error. Statistical differences were determined using unpaired *t-*test. ns, not significant; *P <0.05; **P <0.005; ***P <0.001.

Our study determined that rEFTu bound holo-transferrin and released transferrin-related iron. To detect the interaction between holo-transferrin and EFTu at a whole-bacteria level, we measured the ability of the five BL21 strains carrying a series of pEImC plasmids to bind holo-transferrin and take up transferrin-related iron. The fluorescein-holo-transferrin binding ability of BL21-pEImC-EFTu, BL21-pEImC-ΔTu1, BL21-pEImC-ΔTu2, and BL21-pEImC-ΔTu3 strains was significantly stronger than that of the BL21-pEImC strain (*P* < 0.001, Figure 6A). Compared with the BL21-pEImC-EFTu strain, BL21-pEImC-ΔTu1, BL21-pEImC-ΔTu2, and BL21-pEImC-ΔTu3 strains showed significantly weaker holo-transferrin binding ability, which indicated that all three domains of EFTu were involved in the process of combining holo-transferrin (*P* < 0.001, Figure 6A). To detect the iron acquisition ability of the five BL21 strains carrying a series of pEImC plasmids, calcein fluorescence-quenching assays and holo-transferrin conversion assays were performed. In calcein fluorescence-quenching assays, a lower calcein fluorescence value for the bacteria indicated a stronger iron uptake ability. The iron uptake abilities of BL21-pEImC-EFTu, BL21-pEImC-ΔTu1, BL21-pEImC-ΔTu2, and BL21-pEImC-ΔTu3 were significantly stronger than that of BL21-pEImC strain (*P* < 0.001, Figure 6B). Furthermore, deletion of any domain of EFTu significantly reduced the uptake of transferrin-related iron by bacteria (*P* < 0.001). Holo-transferrin conversion assays showed that relative OD_450_ values of BL21-pEImC-EFTu, BL21-pEImC-ΔTu1, BL21-pEImC-ΔTu2, and BL21-pEImC-ΔTu3 strains were significantly lower than those of the BL21-pEImC strain (*P* < 0.05, Figure 6C). However, compared with BL21-pEImC-EFTu, BL21-pEImC-ΔTu1, BL21-pEImC-ΔTu2, and BL21-pEImC-ΔTu3 strains had significantly reduced relative OD_450_ values (*P* < 0.05, Figure 6C). These results indicated that the overexpression of EFTu promoted bacteria transforming holo-transferrin, and the loss of any domain impairs its transformation ability.

The above results indicated that EFTu was positively correlated with holo-transferrin binding and iron uptake ability at the whole-bacteria level. Each domain of EFTu participated in the holo-transferrin binding and the iron uptake processes.

### Overexpression EFTu promotes the serum and growth abilities of E. coli

**Figure 7.**
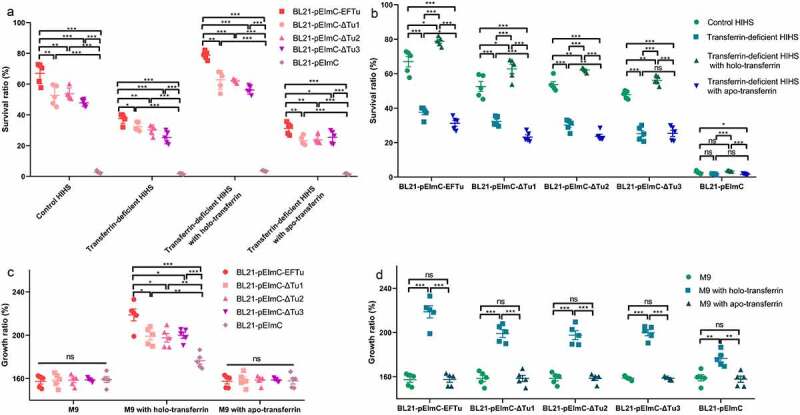
Eftu promotes the viability of *E. coli* in serum and medium by utilizing holo-transferrin. (a-b) BL21-pEimc, BL21-pEimc-EFTu, BL21-pEimc-δtu1, BL21-pEimc-δtu2, and BL21-pEimc-δtu3 strains were incubated with control HIHS, transferrin-deficient HIHS, transferrin-deficient HIHS supplemented with holo-transferrin or apo-transferrin for 3 h. Bacterial survival ratios were calculated as the ratios of the numbers of bacteria recovered from incubation serums to the numbers of original bacteria. (c-d) BL21-pEimc, BL21-pEimc-EFTu, BL21-pEimc-δtu1, BL21-pEimc-δtu2, and BL21-pEimc-δtu3 strains were incubated with M9 medium, M9 medium supplemented with holo-transferrin, or M9 medium supplemented with apo-transferrin for 3 h. Bacterial growth ratios were calculated as the ratios of the numbers of bacteria recovered from incubation medium to the numbers of original bacteria. Data represent mean ± standard error. Statistical differences were determined using unpaired *t* test. ns, not significant; *P <0.05; **P <0.005; ***P <0.001.

This study showed that EFTu of ExPEC had the ability to bind to holo-transferrin, and holo-transferrin promotes the survival ability of ExPEC in HIHS. To explore whether EFTu would enhance the serum tolerance of *E. coli*, we examined the survival ability of the five BL21 strains carrying a series of pEImC plasmids in control HIHS, transferrin-deficient HIHS, and holo-transferrin or apo-transferrin supplemented transferrin-deficient HIHS. The survival ratio of BL21-pEImC-EFTu, BL21-pEImC-ΔTu1, BL21-pEImC-ΔTu2, and BL21-pEImC-ΔTu3 in all kinds of HIHS were significantly higher than that of the BL21-pEImC strain (*P* < 0.001, Figure 7A). However, the survival ratios of BL21-pEImC-ΔTu1, BL21-pEImC-ΔTu2, and BL21-pEImC-ΔTu3 were significantly lower than that of the BL21-pEImC-EFTu strain in all kinds of HIHS (*P* < 0.01, Figure 7A). The survival ratios of BL21-pEImC-EFTu, BL21-pEImC-ΔTu1, BL21-pEImC-ΔTu2, and BL21-pEImC-ΔTu3 strains in the transferrin-deficient HIHS and the apo-transferrin-supplemented transferrin-deficient HIHS were significantly lower than that of the control HIHS and the holo-transferrin-supplemented transferrin-deficient HIHS (*P* < 0.05, Figure 7B). There was no difference in the growth ratios of each pEImC strain in M9 medium, which excluded the influence of the M9 medium on the growth of the bacteria (*P* > 0.05, Figure 7C). The growth ratio of bacteria in M9 medium supplemented with holo-transferrin tended to be consistent with the growth ability in different kinds of HIHS, indicating that holo-transferrin was responsible for the difference in bacterial growth in HIHS (*P* < 0.01, Figure 7D). The growth abilities of the five BL21 strains carrying a series of pEImC plasmids in M9 medium supplemented with apo-transferrin showed no significant difference when compared with those in untreated M9 medium, indicating that apo-transferrin did not cause differences in the survival of the five BL21 strains carrying a series of pEImC plasmids in HIHS (*P* > 0.05, Figure 7D). These results indicated that holo-transferrin in HIHS, but not apo-transferrin, significantly enhanced the serum survival ability of *E. coli* overexpressing EFTu or EFTu domains on the surface.

## Discussion

As a cofactor for diverse fundamental physiological activities, iron is an essential nutrient element for host cells and most pathogenic bacteria. However, most iron is sequestrated by host iron-carrying proteins such as transferrin and lactoferrin, limiting its bioavailability to microorganisms [39]. This iron restriction, termed nutritional immunity, is an effective strategy for host defense against pathogenic infection and an important part of the innate defense system [40].

In normal human serum, free iron is too scarce to support bacterial growth. Transferrin is an abundant serum iron storage protein [41]. The concentration of transferrin in normal human serum ranges from 1 mg/mL to 4 mg/mL, with saturated holo-transferrin accounting for about 30% [42,43]. Therefore, transferrin is a target for pathogens to obtain iron. In a typical example, *Neisseria* utilize TbpA and TbpB on their surface to bind holo-transferrin and release iron, which is transported into the cytoplasm by the TonB transport system [22,44,45]. *M. tuberculosis* utilizes GAPDH on its surface to recruit holo-transferrin and transports holo-transferrin into the cytoplasm [23].

There is sufficient evidence that proteins with typical functions in the bacterial cytoplasm also play an important role on the surface of bacteria [27,46,47]. These proteins are called moonlighting proteins. EFTu is one of the most widely studied moonlighting proteins. EFTu of *P. aeruginosa*, *Leptospira*, *Streptococcus pneumoniae*, and *Mycoplasma hyopneumoniae* binds to factor H and/or plasminogen for immune evasion [12,13,48]. EFTu of nontypeable *Haemophilus influenzae*, *Mycoplasma hyopneumoniae*, and *Streptococcus suis* Type 2 interacts with fibronectin and/or laminin to mediate the adhesion of bacteria to host cells [16,49,50]. EFTu of *Lactobacillus reuteri* mediates adhesion to Caco-2 cells and the mouse gastrointestinal tract [51]. Theses researches on EFTu indicated that EFTu is an important virulence factor. In addition, EFTu can elicit an immune response to protect the host from pathogen infection. For example, immunization with EFTu contributes to protection against infection by *S. pneumoniae*, *H. influenzae*, and *S. suis* 2, indicating that EFTu could be a candidate antigen target for vaccines [52–54].

In this study, we confirmed a novel moonlighting function of EFTu in binding holo-transferrin but not apo-transferrin and releasing transferrin-related iron on the surface of *E. coli*. In the interaction between EFTu and holo-transferrin, EFTu acted like an enzyme, binding the substrate holo-transferrin, catalyzing the conversion of holo-transferrin into the product apo-transferrin, and releasing apo-transferrin. This was a dynamic and efficient process that promoted the acquisition of transferrin-related iron by ExPEC. Unlike the specialized transferrin-binding proteins TbpA and TbpB of *Neisseria*, EFTu of ExPEC acted as a moonlighting protein to play a transferrin-binding role. Moreover, although GAPDH of *M. tuberculosis* was also a moonlighting transferrin-binding protein, EFTu functioned as an enzyme, while GAPDH functioned as a transporter [23].

EFTu was not only located in the cytoplasm, but also expressed on the surface of bacteria. Our study confirmed the surface localization of EFTu on ExPEC. Other studies have reported that EFTu was found in outer membrane vesicles (OMVs) or membrane vesicles (MVs) of Shiga Toxin-Producing *E. coli*, *Burkholderia pseudomallei*, *Acinetobacter baumannii*, and *Bifidobacterium longum* [55–58]. OMVs and MVs possess a variety of functions, including the acquisition of nutrients, transfer of virulence factors and genes, interception of host defensive factors, and modulation of the host immune response [59,60]. The roles of EFTu in OMVs/MVs in the holo-transferrin binding and iron release/uptake processes require further research.

Duplication of the *eftu* gene is widespread in many Gram-negative bacteria [61]. There are two almost identical copies of *eftu* in the *E. coli* genome in different locations. Therefore, deletion of one of the copies does not affect the function of the bacteria. However, due to the essential role of EFTu in protein biosynthesis, deletion of two copies is fatal to bacterial growth. Therefore, we failed to construct an *eftu* knockout strain. In this research, the interaction between EFTu and transferrin was mainly verified through recombinant proteins. In addition, we constructed a surface expression vector to express EFTu on the surface of *E. coli* strain BL21. Through surface expression strains, the interaction between EFTu and holo-transferrin was further demonstrated at the whole-bacteria level. We also confirmed that EFTu promoted the uptake of transferrin-related iron by *E. coli*.

Studies have shown that fragments of EFTu retained the function of binding host proteins [62]. In *Mycoplasma pneumoniae*, EFTu utilizes its carboxyl region to bind to fibronectin [50]. In the interaction between *M. pneumoniae* and factor H, all three domains of EFTu are involved in the binding of factor H [63]. To explore the interaction regions between ExPEC EFTu and holo-transferrin, we constructed a series of recombinant proteins with each domain deleted and confirmed their ability to bind to holo-transferrin and to release iron after binding. Each domain of EFTu contributed to the interaction with holo-transferrin, suggesting that the interaction sites were distributed in all domains. This also suggested that proteins with similar EFTu domains may be potential transferrin-binding and holo-transferrin iron-releasing proteins, which have the ability to promote the uptake of iron by bacteria. The EFTu activity was not abolished in any of the three domain deletion mutants, suggesting that none of the three domains seems indispensable, which indicated that a strict conformational structure of EFTu is not required for holo-transferrin binding or iron release. It also indicated there were some redundancies between the three domains of EFTu, especially regarding the enzyme-like activity of conversion of holo-transferrin to apo-transferrin.

In summary, our research revealed a novel holo-transferrin binding protein of ExPEC. Surface-located EFTu distinguished between holo-transferrin and apo-transferrin, specifically binding holo-transferrin rather than apo-transferrin in a dose-dependent manner. EFTu also promoted the release of transferrin-related iron. All domains of EFTu participated in holo-transferrin binding and iron release. EFTu improved the viability of *E. coli* in serum by acquiring transferrin-related iron. This study provided a novel perspective for research into the molecular mechanism of ExPEC iron acquisition and pathogenicity.

## Supplementary Material

Supplemental Material

## Data Availability

The datasets used and/or analyzed during the current study are available from the corresponding author on reasonable request.
