# Supplementary material for: Extraintestinal pathogenic Escherichia coli utilizes the surface-expressed elongation factor Tu to bind and acquire iron from holo-transferrin
Source: Virulence. 2022 Apr 20;13(1):698–713. doi: 10.1080/21505594.2022.2066274 (PMC9037478; doi:10.1080/21505594.2022.2066274)
Supplement: Supplemental Material [file KVIR_A_2066274_SM4768.pdf]

Supplementary file

Supplementary Materials and Methods

Apo-transferrin was diluted to different concentrations (0.1, 1, 10, 100 μg/mL) with coating solution. Then 100 μL per well was coated on ELISA plate overnight at 4°C. Wells coating without proteins worked as blank control. After blocking with BSA, the ELISA plate was incubated with the anti-transferrin antibody (1:10000) for 2 h at room temperature. Wells were washed with PBST for five times. Then wells were incubated with HRP-conjugated anti-rabbit IgG antibody for 1 h and washed with PBST for five times. According to the manufacturer's instructions, TMB solution (TIANGEN, Catalog No. PA107) was added to each well, followed by the addition of stop solution (2 M H_2SO_4). The OD_{450} values were measured using a Tecan Spark Reader. This assay repeated four times.

Supplementary Results

To test whether the anti-transferrin antibody reacts with apo-transferrin, the ELISA assay of apo-transferrin was performed. As Fig. S1 showed, the OD_{450} value of the apo-transferrin -coated wells has no significant difference with the OD_{450} value of the blank control wells ($P < 0.05$), indicating that the apo-transferrin cannot be detected by the anti-transferrin antibody.

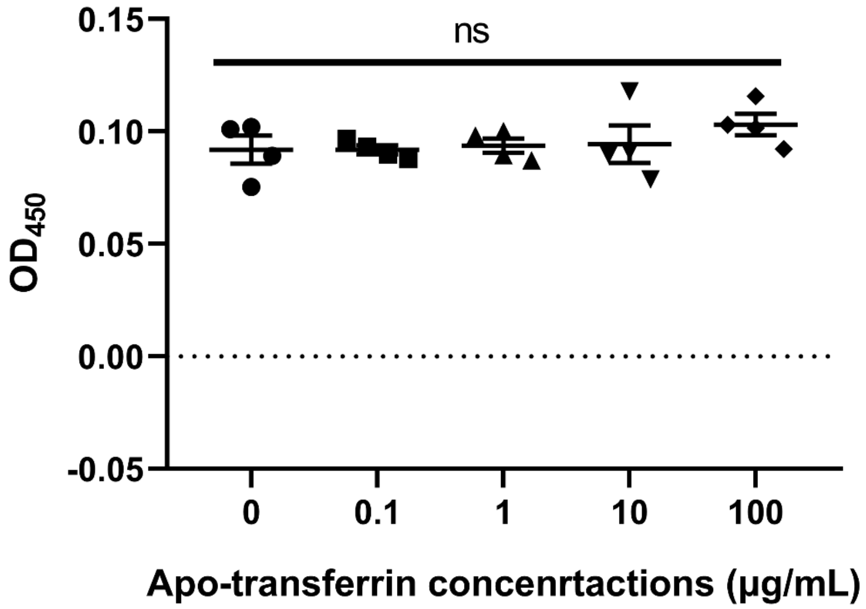

Figure S1. The ELISA assay of apo-transferrin. Different concentrations of

apo-transferrin were coated on ELISA plate. After blocking, the ELISA plate was incubated with the anti-transferrin antibody. The OD_{450} values were obtained by incubating with HRP-conjugated anti-rabbit IgG. Data are expressed as the mean ± standard error. Statistical differences were determined using unpaired *t* test. ns, not significant.
